# Long-term outcomes of conventional and novel steroid replacement therapy on bone health in primary adrenal insufficiency

**DOI:** 10.1038/s41598-022-13506-5

**Published:** 2022-08-02

**Authors:** Valentina Guarnotta, Claudia Di Stefano, Carla Giordano

**Affiliations:** Dipartimento di Promozione della Salute, Materno - infantile, Medicina Interna e Specialistica di Eccellenza “G. D’Alessandro” (PROMISE), Sezione di Malattie Endocrine, del Ricambio e della Nutrizione, Università di Palermo, piazza delle cliniche 2, 90127 Palermo, Italy

**Keywords:** Endocrinology, Health care

## Abstract

Steroids affect bone health causing osteoporosis and fractures. The study aims to compare dual-release hydrocortisone (DR-HC) and conventional steroids on bone metabolism in patients with primary adrenal insufficiency (PAI). Thirty-five patients with PAI on conventional steroids (group A) and 35 patients switched to DR-HC (group B), consecutively referred at our hospital, were evaluated at baseline and after 18, 36 and 60 months of treatment. After 60 months of follow-up, patients in group A had a significant increase in body mass index (*p* = 0.004) and waist circumference (WC) (*p* = 0.026) and a significant decrease in osteocalcin (*p* = 0.002), bone alkaline phosphatase (*p* = 0.029), lumbar spine bone mass density (BMD) T and Z scores (*p* < 0.001 and *p* = 0.001, respectively) and vertebral fractures rate (*p* = 0.021) than baseline. By contrast, patients in group B had a significant decrease in WC (*p* = 0.047) and increase in bone alkaline phosphatase (*p* = 0.019), lumbar spine BMD T score (*p* = 0.032), femoral neck BMD T and Z scores (*p* = 0.023 and *p* = 0.036, respectively) than baseline. Long-term conventional steroid replacement therapy is associated with a decrease in BMD, notably at lumbar spine, and increase in vertebral fractures rate. By contrast, DR-HC treatment is associated with improvement of BMD.

## Introduction

Steroid-induced osteoporosis is the most common cause of secondary osteoporosis and the most common iatrogenic cause of the disease^1^. Patients with adrenal insufficiency (AI) need chronic steroid replacement therapy, exposing them to an increased risk of comorbidities^2^. Conventional steroid treatment is associated with bone fragility and vertebral fractures^3^. The negative impact of steroids on bone is well known, being related to a number of factors, such as the dose and type of steroid used, the duration of therapy, and the cumulative dose^4^, but also age, white race, female gender, smoking and alcohol can have an impact on bone loss^5^. Generally, steroids can induce bone loss in the short term up to one year of treatment with a loss of bone mass density (BMD) of 6–12% and in the long term with a BMD loss of 3% per year^1^. The risk significantly decreases when the steroid treatment is interrupted^5^. The risk of osteoporosis and fractures is increased for replacement steroid doses more than 30 mg/day of hydrocortisone or equivalent^6^ or for prednisone treatment, even at low doses^7^.

By contrast, novel formulations of hydrocortisone (HC), and specifically dual-release HC (DR-HC), are associated with improvement of BMD^8^.

The objective of the current study was to compare the effects of DR-HC and conventional steroids in patients with primary AI (PAI), on bone metabolism, fracture rate and BMD at lumbar spine and femoral neck during 60 months of follow-up.

## Materials and methods

### Study participants

We evaluated data from 70 consecutive patients with PAI on conventional steroid treatment in a real-life study. Patients were consecutively referred to the Division of Endocrinology of Palermo University from January 2012 to December 2020. Patients were on conventional steroid treatment (cortisone acetate and HC), administered twice or three times a day. Thirty-five patients, 15 males and 20 females, maintained conventional steroid therapy (17 cortisone acetate and 18 HC) (group A) while the other 35, (16 who were on cortisone acetate and 19 who were on HC), 11 males and 24 females, were switched from conventional steroid treatment to DR-HC (group B) administered orally in the morning in a fasting state. Patients had a 60-month follow-up. In group A there were 5 women in menopause, while in group B there were 6 women in menopause. Exclusion criteria were the following: age ≤ 18 years, secondary AI (SAI), treatment with other steroids (prednisone), pregnancy, breastfeeding, premature ovarian failure, hypoparathyroidism, hyperparathyroidism, treatment with estrogens and underweight (BMI < 18.5 kg/m^2^). The switch to DR-HC was judged to be appropriate on clinical grounds in those patients who complained of fatigue and weakness, presented hyponatraemia (< 134 mmol/L) or hypoglycaemia (≤ 2.78 mmol/L) or showed more than two comorbidities such as diabetes, osteoporosis/osteopenia, arterial hypertension and central obesity. The switch from HC to DR-HC was made with an equivalent dose, while the dose was reduced from cortisone acetate to DR-HC taking into consideration the minor steroid activity of cortisone acetate compared to HC and patients’ clinical characteristics.

PAI was diagnosed as recommended by international guidelines^9^.

In detail, among the total of 70 patients, 42 had autoimmune polyglandular syndrome (APS), while 28 had isolated autoimmune AI. Among patients with APS, 26 had combined Addison’s disease and autoimmune thyroid disease, 6 had combined Addison’s disease, type 1 diabetes mellitus and autoimmune hypothyroidism and 10 had combined Addison’s disease, autoimmune hypothyroidism and celiac disease. Patients with celiac disease were on a stable gluten-free diet. All patients with PAI were also on stable treatment with fludrocortisone (0.05–0.1 mg/day, once). Patients with hypothyroidism were treated with levo-thyroxine at the average dose of 1.2–1.5 mcg/kg. Patients with type 1 diabetes were on basal-bolus treatment on flash blood glucose monitoring. Five postmenopausal women had been treated with DHEA for a ranging period of 6–18 months, before being included in the study.

During the 60-month treatment period, the conventional steroid and the DR-HC doses were changed based on the physician’s judgement of a patient’s need in both groups of patients (Table 1). Each patient received instructions for treatment in special or emergency situations. Patients treated with DR-HC were instructed to add a rescue dose of HC during an intercurrent illness or stress (5 or 10 mg according to severity of stress and symptoms). Overall, 8 patients had to take a rescue dose of HC, 5 of them less than 10 times and 3 of them from 20 to 30 times during the 60-month period.

**Table 1:** Dose adjustments according to the physician’s judgement during the 60 months of conventional glucocorticoid and dual-release hydrocortisone treatments.

Baseline dose (N° = 35)	Dose at 60 months of DR-HC				
	20 mg/day	25 mg/day	30 mg/day	35 mg/day	40 mg/day
20 mg/day (no. = 15)	13	1	0	0	1
25 mg/day (no. = 12)	0	10	1	1	0
30 mg/day (no. = 3)	0	0	3	0	0
40 mg/day (no. = 5)	0	0	2	0	3

Baseline dose (N = 17)	Dose at 60 months of cortisone acetate				
	25 mg/day	37.5 mg/day	47.75 mg/day	50 mg/day	62.5 mg/day
25 mg/day (no. = 1)	1	0	0	0	0
37.5 mg/day (no. = 2)	0	2	0	0	0
47.75 mg/day (no. = 1)	0	0	1	0	0
50 mg/day (no. = 8)	0	1	0	7	0
62.5 mg/day (no. = 5)	0	0	0	1	4

Baseline dose (N° = 18)	Dose at 60 months of hydrocortisone				
	15 mg/day	20 mg/day	25 mg/day	30 mg/day	35 mg/day
15 mg/day (no. = 6)	6	0	0	0	0
20 mg/day (no. = 7)	0	6	1	0	0
25 mg/day (no. = 3)	0	0	3	0	0
30 mg/day (no. = 1)	0	0	0	1	0
35 mg/day (no = 1)	0	0	0	0	1

The current study was carried out in accordance with the recommendations of the Paolo Giaccone Policlinico ethics committee, with written informed consent from all subjects. All subjects gave written informed consent in accordance with the Declaration of Helsinki. The protocol was approved by the Paolo Giaccone Policlinico ethics committee (protocol 06/2021).

### Study design

At baseline and after 18, 36 and 60 months of conventional steroid and DR-HC treatment, clinical and bone metabolic parameters were evaluated.

Anthropometric parameters such as BMI and waist circumference (WC), measured at the midpoint between the lower rib and the iliac crest, were evaluated. In addition, sodium, potassium, serum 25hydroxyvitamin-D (vitaminD), parathyroid hormone, calcium, phosphorus, creatinine, osteocalcin and bone alkaline phosphatase were assayed.

The blood sample was taken about 2 h after steroid administration (patients took the dose in the morning on waking) to avoid patients experiencing fatigue or other symptoms due to delayed intake of the drug.

In both groups, hypovitaminosis D was observed at baseline and a pharmacological supplementation was started in 26 patients of group A and 25 of group B, at the mean dose of 800 UI/day and maintained during the follow-up. Hypovitaminosis D was defined as a serum 25-hydroxy vitamin D level below the normal range (< 30 ng/ml). All patients supplemented with vitamin D reached the threshold of 30 ng/ml.

BMD was measured by DXA at lumbar spine and femoral neck (Hologic Horizon Inc., QDR-4500 W Waltham, MA) at baseline and after 18, 36 and 60 months of follow-up.

In patients aged 50 or more, BMD was expressed as the T-score, comparing the results with those obtained in a sex-matched Caucasian population at the peak of bone mass. A T-score less than or equal to − 2.5 SD at the neck or spine was defined as osteoporosis, whereas osteopenia was defined as a T-score between − 1 and − 2.5 SD. In patients younger than 50 years, the results were expressed as a/the Z-score, comparing the results with those obtained in an age and sex-matched Caucasian population. A Z-score of − 2.0 SD or lower was used to define a BMD “below the expected range for age”^10^. The coefficients of variation in the DXA measurements for BMD, bone mineral content (BMC) and area were 0.61%, 2.98% and 2.89%, respectively.

We also evaluated rib, femoral neck and hip fractures rate during the follow-up in both groups.

### Assays

Sodium, potassium, serum 25hydroxyvitamin-D (vitamin-D), parathyroid hormone, calcium, phosphorus, creatinine, osteocalcin and bone alkaline phosphatase were measured with standard methods (Modular P800, Roche, Milan) at our hospital centralized laboratory. The intra- and interassay coefficients of variation were the following: Sodium 0.5% to 1.2% and 1.28% to 1.44%, respectively; potassium 1% to 2.1% and 2.2% to 3.08%, respectively; vitaminD 0.9% to 1.6% and 1.7% to 2.56%, respectively, parathyroid hormone 1.2% to 2.6% and 2.8% to 3.9%, respectively; calcium 1.8% to 3.5% and 3.75% to 4.23%, respectively; phosphorus 1.6% to 3.3% and 3.5% to 4.2%, respectively; creatinine 1.7% to 2.9% and 3.3% to 4.6%, respectively; osteocalcin 1.9% to 3.3% and 3.6% to 4.9%, respectively; bone alkaline phosphatase 2.7% to 3.6% and 3.8% to 5.2%, respectively.

### Statistical analysis

The Statistical Packages for Social Science SPSS version 19 (SPSS, Inc., IBM, New York, USA) were used for data analysis. The normality of quantitative variables was tested with the Shapiro-Wilk test. The baseline characteristics of the groups were presented as mean ± SD for continuous variables, while the rates and proportions were calculated for categorical data. The differences between groups were performed using ANOVA for quantitative variables and the χ^2^-test for categorical variables. A comparison between numerical variables at baseline, 18-, 36- and 60-month follow-up was performed with the Friedman analysis. In addition, multiple linear regression analysis was performed to identify independent predictors of the dependent variables lumbar spine and femoral neck T and Z scores and of dependent variable fracture. A *p* value < 0.05 was considered statistically significant.

## Results

The baseline characteristics of all patients with PAI and subgroups (A and B) are shown in Table 3. During the 60-month period of observation, 4 out of 35 patients had an adrenal crisis in group A and none in the group B.

At baseline, patients in group B had a higher frequency of osteoporosis/osteopenia (*p* = 0.001) and visceral obesity (*p* = 0.020), higher WC values (*p* = 0.048) and lower osteocalcin levels (*p* = 0.004) than group A (Table 2).

**Table 2:** General characteristics of all patients and subgroups A and B at baseline.

	All baseline (No. = 70)	Group A baseline (No. = 35)	Group B baseline (No. = 35)	p*
	Subjects (%)	Subjects (%)	Subjects (%)	
**Gender**				
Male	26 (37.1%)	15 (42.8%)	11 (31.4%)	0.462
Female	44 (62.8%)	20 (57.1%)	24 (68.5%)	
Arterial hypertension	12 (17.1%)	5 (21.3%)	7 (20%)	0.488
Osteoporosis/osteopenia	20 (28.5%)	3 (8.5%)	17 (48.5%)	0.001
Rib fractures	2 (2.8%)	0	2 (5.7%)	0.403
Femoral neck fractures	1 (1.4%)	0	1 (2.8%)	0.556
Hypovitaminosis D	51 (71.4%)	26 (74.2%)	25 (71.4%)	0.924
Bone resorption inhibitors	17 (24.2%)	5 (14.3%)	11 (31.4%)	0.297
Visceral obesity	37 (52.8%)	12 (34.3%)	25 (71.4%)	0.020
Hypercholesterolemia	11 (15.7%)	2 (5.7%)	9 (25.7%)	0.220
Diabetes mellitus	14 (20%)	4 (11.4%)	10 (28.5%)	0.425
**Replacement therapy**				
Cortisone acetate	35 (50%)	17 (48.5%)	18 (51.5%)	0.479
Hydrocortisone	35 (50%)	18 (51.5%)	17 (48.5%)	

	Mean ± SD	Mean ± SD	Mean ± SD	
Duration of disease (yrs)	14.7 ± 11.6	14.7 ± 12.2	14.9 ± 10.8	0.942
Age (yrs)	49.7 ± 22.1	50.9 ± 17.4	49.5 ± 13.4	0.754
**Anthropometric parameters**				
BMI (Kg/m^2^**)**	26.4 ± 5.31	26.2 ± 5.94	27.3 ± 5.71	0.679
Waist circumference (cm)	96.2 ± 13.2	94.2 ± 12.3	98.7 ± 13.8	0.048
**Electrolytes**				
Na (mmol/L)	136.3 ± 3.71	137.7 ± 0.97	136.1 ± 3.21	0.326
K (mmol/L)	4.63 ± 0.51	4.31 ± 0.75	4.56 ± 0.47	0.399
**Bone metabolic parameters**				
Calcium (mg/dL)	9.61 ± 0.58	10.1 ± 0.65	9.51 ± 0.51	0.073
Phosphorus (mg/dL)	3.86 ± 0.68	4.06 ± 0.37	3.72 ± 0.71	0.420
Parathyroid hormone (pg/mL)	48.3 ± 33.1	54.4 ± 20.7	75.3 ± 15.8	0.788
VitaminD (ng/ml)	17.3 ± 3.61	18.1 ± 8.58	16.4 ± 5.51	0.600
Creatinine (mg/dL)	0.91 ± 0.43	1.04 ± 0.82	0.85 ± 0.19	0.239
Osteocalcin (ng/mL)	34.6 ± 12.8	32.4 ± 9.87	23.7 ± 14.7	0.004
Bone alkaline phosphatase (U/L)	74.4 ± 32.7	62.7 ± 20.2	58.4 ± 32.7	0.296
Lumbar spine (L1-L4) BMD T score	− 1.29 ± 1.23	− 0.96 ± 0.84	− 2.14 ± 1.46	0.028
Lumbar spine (L1-L4) BMD Z score	− 0.48 ± 0.85	− 0.16 ± 0.58	− 0.94 ± 0.92	0.025
Femoral neck BMD T score	− 1.71 ± 1.44	− 1.2 ± 0.76	− 1.59 ± 1.74	0.093
Femoral neck BMD Z score	− 0.67 ± 1.03	− 0.61 ± 0.59	− 0.76 ± 0.51	0.740

*p value between patients in group A and B at baseline.

After 60 months of follow-up, patients in group A had a significant increase in BMI (*p* = 0.004), WC (*p* = 0.026), vitamin D (*p* = 0.005), and a significant decrease in osteocalcin (*p* = 0.002), bone alkaline phosphatase (*p* = 0.024), lumbar spine BMD T and Z scores (*p* < 0.001 and *p* = 0.001, respectively) compared to baseline (Table 3). By contrast, patients in group B had a significant decrease in WC (*p* = 0.047) and increase in vitamin D (*p* < 0.001), bone alkaline phosphatase (*p* = 0.019) lumbar spine BMD T score (*p* = 0.032), femoral neck BMD T and Z scores (*p* = 0.023 and *p* = 0.036, respectively) compared to baseline (Table 3). In addition, at 60 months of follow-up we observed significantly higher values of WC (*p* = 0.045) and bone alkaline phosphatase (*p* < 0.001) and significantly lower values of osteocalcin (*p* = 0.001), lumbar spine and femoral neck Z scores (*p* = 0.045 and *p* = 0.047, respectively) in group A compared to group B (Table 3).

**Table 3:** Anthropometric and metabolic parameters in patients of groups A and B with primary adrenal insufficiency (PAI) at baseline and after 60 months of treatment.

	PAI Group A (No. = 35)		p*	PAI Group B (No. = 35)		p**	p***
	Baseline Mean ± SD	60 months Mean ± SD		Baseline Mean ± SD	60 months Mean ± SD		
**Anthropometric parameters**							
BMI (Kg/m^2^)	26.2 ± 5.94	28.5 ± 5.81	0.004	27.3 ± 5.71	26.3 ± 4.09	0.098	0.083
WC (cm)	94.2 ± 12.3	101.2 ± 13.8	0.026	98.7 ± 13.8	96.1 ± 13.4	0.047	0.045
**Bone metabolic parameters**							
Calcium (mg/dL)	9.91 ± 0.53	9.28 ± 0.56	0.056	9.65 ± 0.68	9.51 ± 0.51	0.410	0.403
Phosphorus (mg/dL)	3.93 ± 0.51	3.89 ± 0.69	0.244	4.05 ± 0.72	3.69 ± 0.68	0.180	0.240
Parathyroid hormone (pg/mL)	48.7 ± 24.1	46.7 ± 23.5	0.828	50.5 ± 43.5	47.2 ± 13.2	0.762	0.470
Vitamin D (ng/mL)	18.3 ± 9.55	37.4 ± 8.19	0.005	16.4 ± 5.51	37.8 ± 5.41	< 0.001	0.505
Creatinine (mg/dL)	0.76 ± 0.11	0.75 ± 0.16	0.689	0.83 ± 1.38	0.76 ± 0.06	0.510	0.183
Osteocalcin (ng/mL)	32.4 ± 9.87	18.6 ± 11.5	0.002	23.7 ± 14.7	34.7 ± 7.86	0.139	0.001
Bone alkaline phosphatase (U/L)	62.7 ± 20.2	53 ± 13.8	0.024	58.4 ± 32.7	72.5 ± 26.8	0.019	< 0.001
Lumbar spine (L1-L4) BMD T score	− 0.96 ± 0.84	− 2.08 ± 0.93	< 0.001	− 2.14 ± 1.46	− 1.48 ± 1.65	0.032	0.085
Lumbar spine (L1-L4) BMD Z score	− 0.16 ± 0.58	− 1.31 ± 0.83	0.001	− 0.94 ± 0.92	− 0.44 ± 0.54	0.067	0.045
Femoral neck BMD T score	− 1.21 ± 0.76	− 1.71 ± 1.16	0.050	− 1.59 ± 1.74	− 0.87 ± 1.74	0.023	0.487
Femoral neck BMD Z score	− 0.61 ± 0.59	− 0.96 ± 0.79	0.689	− 0.76 ± 0.51	− 0.27 ± 1.21	0.036	0.047

	Subjects (%)	Subjects (%)		Subjects (%)	Subjects (%)		
Osteoporosis/Osteopenia	3 (14.3%)	11 (31.4%)	0.091	15 (42.8%)	15 (42.8%)	1	0.327
Rib fractures	0	5 (14.2%)	0.183	2 (5.7%)	2 (5.7%)	1	0.550
Neck fractures	0	1 (2.8%)	0.556	1 (2.8%)	1 (2.8%)	1	1
Hip fractures	0	2 (5.7%)	0.403	0	0	1	0.403

*p: comparison between baseline and 60 months of treatment in group A.

**p: comparison between baseline and 60 months of treatment in group B.

***p: comparison between groups A and B at 60 months of treatment.

**Figure 1:**
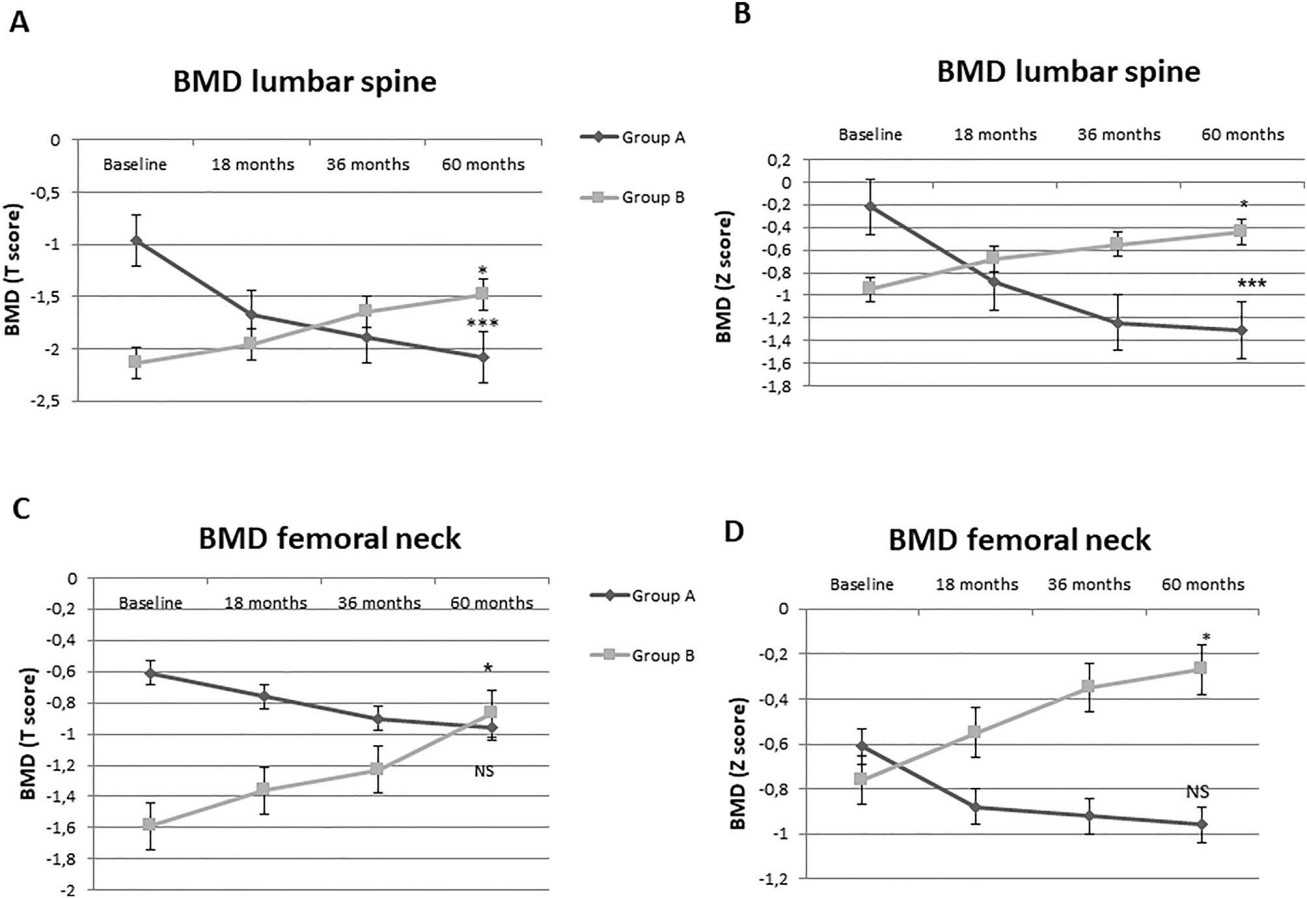
(**A**) Changes in BMD lumbar spine T score from baseline to 60 months for patients of groups A and B. *p < 0.05, **p < 0.01; ***p < 0.001 versus baseline using the Friedman analysis. Data are means (S.D.). (**B**) Changes in BMD lumbar spine Z score from baseline to 60 months for patients of groups A and B. *p < 0.05, **p < 0.01; ***p < 0.001 versus baseline using the Friedman analysis. Data are means (S.D.). (**C**) Changes in BMD femoral neck T score from baseline to 60 months for patients of groups A and B. *p < 0.05, **p < 0.01; ***p < 0.001 versus baseline using the Friedman analysis. Data are means (S.D.). (**D**) Changes in BMD femoral neck Z score from baseline to 60 months for patients of groups A and B. *p < 0.05, **p < 0.01; ***p < 0.001 versus baseline using the Friedman analysis. Data are means (S.D.).

A significant trend of decrease in BMD lumbar spine T and Z scores was observed in group A, while in group B we observed a significant trend of increase during the follow-up (Fig. 1A,B). In addition, a significant trend of increase was also observed in the BMD femoral neck T and Z scores of group B during the follow-up, while no differences were observed in group A (Fig. 1C,D).

**Figure 2:**
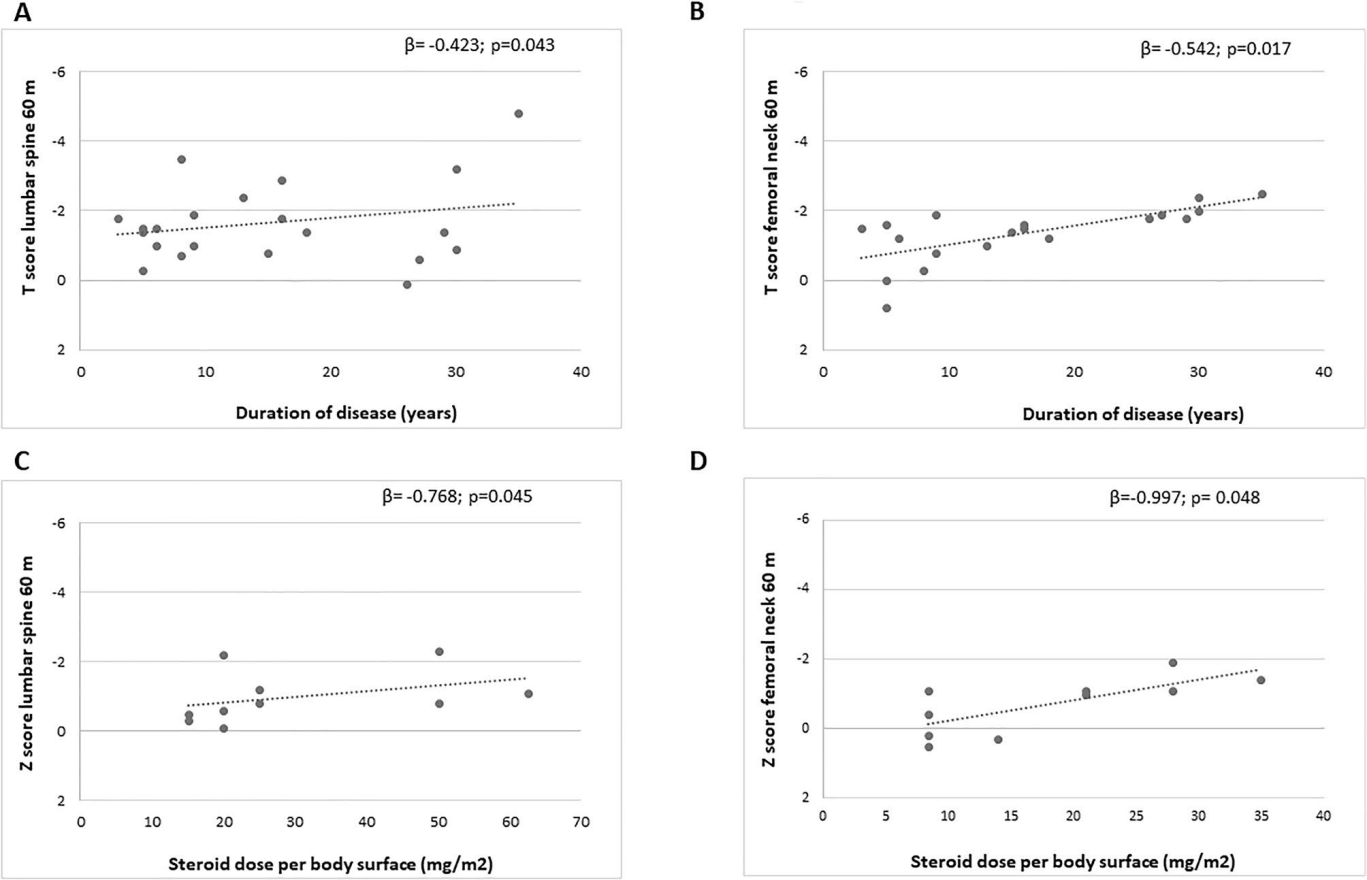
(**A**) Correlation between T score at lumbar spine and duration of disease at 60 months of follow-up. (**B**) Correlation between T score at femoral neck and duration of disease at 60 months of follow-up. (**C**) Correlation between Z score at lumbar spine and steroid daily dose per body surface at 60 months of follow-up. (**D**) Correlation between Z score at femoral neck and steroid daily dose per body surface at 60 months of follow-up.

Multivariate analysis showed that the duration of disease was negatively independently associated with T scores of femoral neck (β = − 0.542, *p* = 0.017) and lumbar spine (β = − 0.423; *p* = 0.043) at 60 months. The daily steroid dose per body surface was negatively independently associated with Z scores of femoral neck (β = − 0.997; *p* = 0.048) and lumbar spine (β = − 0.768; *p* = 0.045) at 60 months (Fig. 2). The rate of vertebral fractures in group A was also positively independently associated with daily steroid dose per body surface (β = 0.875; *p* = 0.048) (Table 4).

**Table 4:** Variables independently associated with the fracture rate after 5 years of conventional steroid replacement therapy (group A) at multivariate analysis.

	Fracture rate after 5 years of treatment			
	*β*	SE	95%CI	*p* value
Duration of disease	0.271	0.125	− 0.345 to 0.987	0.181
Steroid dose per body surface	0.875	0.183	− 0.105 to 1.252	0.048
Lumbar spine (L1-L4) BMD T score	-0.393	0.278	− 1.035 to 0.249	0.196
Lumbar spine (L1-L4) BMD Z score	0.034	0.201	− 0.429 to 0.498	0.868
Femoral neck BMD T score	0.093	0.188	− 0.339 to 0.526	0.632
Femoral neck BMD Z score	0.485	0.299	− 0.204 to 1.175	0.142

## Discussion

In the current study, we demonstrated that patients on conventional steroid treatment had a significant decrease in lumbar spine BMD T and Z scores, with increased rate of fractures in the 5-year period of observation. This resulted in a significant association between daily steroid dose per body surface and BMD and between duration of disease and BMD, while fracture rate was associated with the steroid dose per body surface. By contrast, DR-HC was associated with improvement in BMD and no new cases of fractures.

Steroids induce osteoporosis by several mechanisms such as inhibition of osteoblast activity, stimulation of osteoclast activity and inhibition of calcium absorption, stimulating renal excretion^11^. Steroids act not only by decreasing BMD, but also by altering the structure of the bone, favouring the risk of fracture^12^. Generally, there is a rapid fall in BMD due to bone resorption, followed by a slow decline due to altered bone formation^5^. Conventional steroid replacement therapy administered in two or three daily doses is characterized by a peak-to-trough release profile with inevitable supraphysiological cortisol values, notably in the evening hours, affecting bone formation and resorption.

The steroid effects on BMD and fractures in patients with AI have been investigated, providing conflicting results. The first studies conducted on patients with AI did not report changes in BMD in patients on long-duration treatment with steroids^13,14^. Other studies suggested a gender difference in BMD values, unfortunately providing conflicting results^15–17^. Braatvetdt et al. did not report differences between patients with AI and healthy controls, even though they showed a negative correlation among BMD, steroid dose and duration of disease, in line with our results^18^.

Several recent studies have investigated the relationship between steroid dose and BMD in adrenal insufficiency. Koetz et al. did not show changes in BMD in 81 patients with PAI and 41 with congenital adrenal hyperplasia (CAH) treated with low doses of steroids (mean equivalent hydrocortisone dose of 21.9 mg/day)^19^. Lovas et al. reported a decrease in BMD in patients with PAI treated with equivalent HC dose of 30 mg/day or more compared to healthy controls^20^. Schulz et al. conducted a prospective study on 57 subjects with PAI and 33 with CAH evaluating three different schemes of HC treatment, patients who maintained unchanged dose of HC (mean dose 25 mg/day), patients who increased the HC dose (from a mean of 18.7 to 25.2 mg/day) and patient who decreased the HC dose (from a mean of 30.8 to 21.4 mg/day). The authors showed that after 2 years, those patients who reduced the mean HC dose had an improvement in BMD, while patients with increased dose of HC had a decrease in BMD^21^. Zelissen et al. showed a linear decrease in BMD with the increasing of HC dose in men^17^. By contrast, another study did not report any correlation between reduced spinal BMD and weight-adjusted HC dose in 28 patients with PAI^22^. Further, Danilowicz et al. and Chikada et al. reported no changes in BMD with low HC doses of 10–15 mg in patients with SAI^23,24^. In addition, patients treated with prednisolone showed lower BMD values compared to the other conventional steroid therapies^25^. Overall, these studies showed that high doses of HC (more than 25 mg/day) were associated with reduced BMD, while low doses of HC (less than 25 mg/day) did not affect BMD.

With regard to the correlation between fractures and steroids, a large study on 3129 patients with PAI conducted from 1964 to 2006 reported 221 hip fractures, notably in the first year of observation, independently from age, sex and coexistence of other autoimmune disorders^26^. Camozzi et al. showed a 3–4 fold risk increase of vertebral fractures in 87 patients with PAI compared to healthy controls^27^. Authors did not observe any correlation with BMD, while reported that long duration of disease and supra-physiological doses of steroids were likely associated with fractures. Recently, a metanalysis on 7 studies and a systematic review on 17 studies on fracture rate in patients with CAH, SAI and PAI showed an increased overall risk of fractures compared to healthy controls with a slight and not significant correlation between steroid equivalent dose and osteoporotic fracture rate^28^. However, this metanalysis had several limitations due to the heterogeneity of the studies included.

DR-HC has been shown to ensure a more physiological release of HC due to its pharmacokinetics, with favourable effects on glucose metabolism, body weight, low-grade inflammation and immune response, and reducing the adverse metabolic effects and the related mortality risk frequently observed in patients chronically treated with conventional steroids^29–31^. The switch from conventional steroids to DR-HC is associated with resynchronization of clock genes and restoration of expression of many genes involved in inflammation, immune response, adipogenesis, and oxidative stress^32,33^. Only one study investigated the effects of DR-HC on BMD, showing a significant improvement in BMD values at lumbar spine and femoral neck after 24 months of treatment. However, this study had a retrospective design and was conducted in a small sample of patients with SAI^8^.

The limitations of the current study are the following: first, the lack of randomization in assignment of patients to group A or group B; second, the small sample of patients enrolled due to the rarity of the disease; third, most of the patients enrolled in the study had combined autoimmune deficiencies which could play an adjunctive negative role on bone health, even though the other autoimmune disorders (hypothyroidism, celiac disease and type 1 diabetes mellitus) were all well controlled; forth, higher frequency of osteoporosis in patients of group B than group A could be a bias of the study. However, we excluded patients with premature ovarian failure to avoid any interference from gonadal hormones on bone. Lastly, we did not evaluate genetic polymorphisms in P-glycoproteins, 11-B-hydroxylase and glucocorticoid receptors, which could have an influence on BMD. The strength of the study was the comparison of the effects of two different schemes of steroid replacement treatment for PAI on bone health and metabolism, the conventional one subdivided into two or three daily doses, and the novel one administered once daily. To our knowledge, this has never been done before.

In conclusion, our study shows that in patients with PAI, 5-years conventional steroid replacement therapy is associated with a significant decrease in lumbar BMD T and Z scores and increase in vertebral fractures rate. In these patients, the decrease in lumbar BMD is associated with long duration of disease and high daily steroid dose per body surface, while the increased rate of vertebral fractures is associated with the daily steroid dose per body surface. By contrast, patients treated with DR-HC treatment show an improvement of femoral neck T and Z scores and lumbar T score. Our findings suggest that DR-HC having a physiological glucocorticoid profile release do not affect bone physiology.

However, further larger randomized studies are required to confirm our data.
